# Ascl1 (Mash1) Defines Cells with Long-Term Neurogenic Potential in Subgranular and Subventricular Zones in Adult Mouse Brain

**DOI:** 10.1371/journal.pone.0018472

**Published:** 2011-03-31

**Authors:** Euiseok J. Kim, Jessica L. Ables, Lauren K. Dickel, Amelia J. Eisch, Jane E. Johnson

**Affiliations:** 1 Department of Neuroscience, University of Texas Southwestern Medical Center, Dallas, Texas, United States of America; 2 Department of Psychiatry, University of Texas Southwestern Medical Center, Dallas, Texas, United States of America; Institut de la Vision, France

## Abstract

Ascl1 (Mash1) is a bHLH transcription factor essential for neural differentiation during embryogenesis but its role in adult neurogenesis is less clear. Here we show that in the adult brain Ascl1 is dynamically expressed during neurogenesis in the dentate gyrus subgranular zone (SGZ) and more rostral subventricular zone (SVZ). Specifically, we find Ascl1 levels low in SGZ Type-1 cells and SVZ B cells but increasing as the cells transition to intermediate progenitor stages. In vivo genetic lineage tracing with a tamoxifen (TAM) inducible *Ascl1^CreERT2^* knock-in mouse strain shows that Ascl1 lineage cells continuously generate new neurons over extended periods of time. There is a regionally-specific difference in neuron generation, with mice given TAM at postnatal day 50 showing new dentate gyrus neurons through 30 days post-TAM, but showing new olfactory bulb neurons even 180 days post-TAM. These results show that Ascl1 is not restricted to transit amplifying populations but is also found in a subset of neural stem cells with long-term neurogenic potential in the adult brain.

## Introduction

Adult neural stem cells generate new neurons in the subgranular zone (SGZ) of the hippocampal dentate gyrus and the subventricular zone (SVZ) adjacent to the lateral ventricle [1]. Although Nestin^+^/GFAP^+^ astrocytic Type-1 cells in the SGZ or B cells in the SVZ are considered to be ‘slowly dividing’ stem-like cells that self-renew and generate neurons throughout life [1], the molecular identity of neural stem cells remains incompletely defined. To understand how neural stem cells balance their self-renewal and differentiation in vivo, it is essential to identify intrinsic factors that define neural stem cell populations.

Transcription factors have central roles in regulating stem cell dynamics and reprogramming between distinct somatic lineages [2], [3], [4]. Ascl1, for example, is essential during embryogenesis for neural differentiation [5], is homologous to proneural genes in *Drosophila* [6], and functions counter to Notch signaling to balance progenitor and differentiation states [5]. In addition, Ascl1 is a key factor in reprogramming fibroblasts directly to functional neurons in vitro [3]. The importance of Ascl1 to embryonic neural development makes it a strong candidate for playing a role in adult neurogenesis as well. Indeed, previous studies using a BAC transgenic strain expressing CreER in Ascl1 cells showed Ascl1^+^ cells are largely transit-amplifying progenitors in the SGZ and SVZ, and become postmitotic neurons within 30 days [7]. Here we examine more closely the expression of endogenous Ascl1 in the adult mouse brain and analyze Ascl1 lineage cells utilizing a new knock-in mouse strain, *Ascl1^CreERT2^*. Our results show that Ascl1 is present in the neurogenic lineage earlier than previously reported, and that Ascl1 lineage cells have long-term neurogenic potential in both the SGZ and SVZ in the adult mouse brain. These findings have fundamental implications for our understanding of the molecular identity of the neural stem cell in the postnatal and adult brain.

## Results

### Ascl1 is present in Type-1 and Type-2a cells in the dentate gyrus SGZ of adult mice

**Figure 1:**
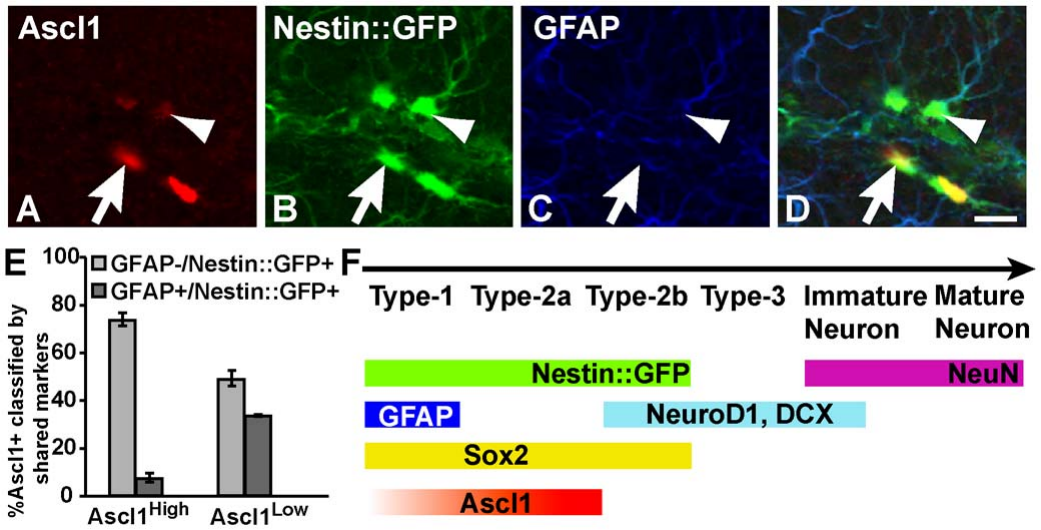
Ascl1 is present in a subpopulation of Type-1 stem cells and Type-2 progenitors in adult hippocampus. (A–D) Ascl1 is weakly detected in Nestin::GFP^+^GFAP^+^ Type-1 stem cells (arrowhead) or strongly detected in Nestin::GFP^+^GFAP^−^ Type-2 progenitors (arrow) in SGZ of adult *Nestin::GFP* mice. (E) Percentage of Ascl1^High^ or Ascl1^Low^ cells that express the markers Nestin::GFP and GFAP (Type-1) (dark shaded bars) or just the marker Nestin::GFP (Type-2) (grey shaded bars). 50 Ascl1^+^ cells were counted per mouse, n = 3 *Nestin::GFP* mice. (F) Ascl1 is in Type-1 and early Type-2 cells based on a current model of adult hippocampal neurogenesis [14]. Scale bar = 20 µm.

Although Ascl1 has been suggested to be a key transcription factor controlling stem cell dynamics [3], [4] in vivo expression of Ascl1 in adult neural stem cell populations has not been thoroughly characterized. To gain a more precise understanding of when Ascl1 is expressed during the stages of adult neurogenesis [14], brain tissue from 8-week old *Nestin::GFP* mice [13] was stained for GFP, GFAP, and Ascl1. Ascl1^+^ cells were easily identified in the adult mouse SGZ (Fig. 1A), as were cells that were categorized as Type-1 (GFAP^+^/Nestin::GFP^+^ and radial glial morphology, Fig. 1B) or Type-2 (GFAP^−^/Nestin::GFP^+^ and progenitor morphology, Fig. 1B). However, it was also evident that Ascl1 cells were heterogeneous in their fluorescent intensity, with some cells expressing high versus low levels of Ascl1 immunoreactivity (Ascl1^High^ versus Ascl1^Low^) (Fig. 1A). Phenotypic analysis revealed that Ascl1^Low^ cells were Type-1 and Type-2, whereas most Ascl1^High^ cells were Type-2 (Fig. 1A–E, arrowheads). Thus, Ascl1 levels generally appear to increase as progenitors are selected for neuronal differentiation (Fig. 1F), a pattern opposite to cells with active Notch signaling as recently reported [15]. This is reminiscent of the *Drosophila* homologs Achaete and Scute that function to select the sensory mother cell from a proneural cluster [16]. Ascl1 may also be expressed in an oscillatory manner as a Notch pathway component [17], a possibility that cannot be determined with static images obtained with immunofluorescence.

**Figure 2:**
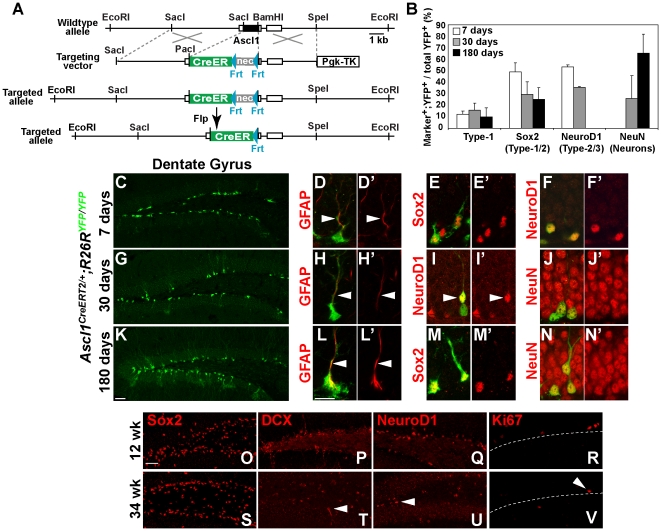
A subset of Ascl1 lineage cells continue to produce new granule neurons 30 days after initial Ascl1 expression in adult hippocampus. (A) Targeting strategy for *Ascl1^CreERT2/+^* knock-in mice. (B) Quantification of the percentage of YFP^+^ cells co-labeled with stage-specific markers in hippocampus of adult *Ascl1^CreERT2/+^;R26R^YFP/YFP^* mice 7, 30, or 180 days post-TAM. 150–500 YFP^+^ cells per mouse were counted for each marker, n = 2 *Ascl1^CreERT2/+^;R26R^YFP/YFP^* mice per time point. (C–F′) 7 days post-TAM YFP^+^ cells co-express GFAP (and have Type-1 morphology), Sox2, or NeuroD1, but not NeuN. (G–J′) 30 days post-TAM YFP^+^ cells overlap with NeuN, but also can co-express GFAP or NeuroD1. (K–N′) 180 days post-TAM a subpopulation of YFP^+^ cells are still Type-1 cells by morphology and express GFAP and Sox2, whereas the majority of YFP^+^ cells express NeuN but not NeuroD1. (O–V) Neurogenesis in the SGZ dramatically decreases between 12 weeks and 34 weeks of age as seen in the decrease in DCX (P,T), NeuroD1 (Q,U) and Ki67 (R,V). Arrowheads indicate the few cells positive for these markers in the 34 week old mice. Notably, Sox2 does not decrease (O,S) so may label quiescent Type-1 like cells. Scale bars  = 50 µm (C,G,K), 10 µm (D–F′, H–J′, I–V).

**Figure 3:**
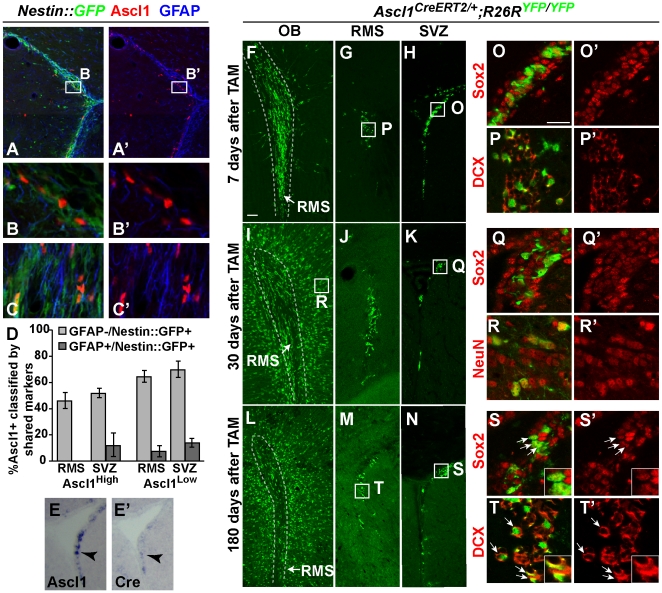
A subset of Ascl1 lineage cells in adult SVZ have long term self renewing properties in the generation of olfactory bulb neurons. (A–D) Ascl1 is detected in Nestin::GFP^+^GFAP^+^ cells (B cells) in the SVZ (A–B′) and in Nestin::GFP^+^GFAP^−^ C cells in SVZ (A–B′) and RMS (C,C′) in 8 week old *Nestin::GFP* transgenic mice. (D) Percentage of Ascl1^High^ or Ascl1^Low^ cells that express the markers Nestin::GFP and GFAP (dark shaded bars) or just Nestin::GFP (grey shaded bars) in the RMS and the SVZ. 25 Ascl1^+^ cells per mouse were counted in the RMS; 60 Ascl1^+^ cells per mouse in the SVZ, n = 3 *Nestin::GFP* mice. (E–E′) mRNA in situ with Ascl1 (E) or Cre (E′) probes in the adult SVZ. (F–T′) Immunofluorescence in *Ascl1^CreERT2/+^*;*R26R^YFP/YFP^* mouse brain sections harvested 7, 30, or 180 days post-TAM demonstrates Ascl1 derived cells along the SVZ-RMS-OB pathway (F–N). 7 days post-TAM most YFP^+^ cells were located in the SVZ, or along the RMS (F–H) and express Sox2 (O–O′) or DCX (P–P′). 30 or 180 days post-TAM YFP^+^ cells mature into neurons in the granule cell layer or the periglomerular layer of the OB (I, L, R–R′, and data not shown). However, many YFP^+^ cells remain as Sox2^+^ or DCX^+^ progenitors in the RMS or SVZ (J–K, M–N, Q–Q′, S–T′). Scale bars = 50 µm (F–N), 10 µm (O–T′).

These expression data place Ascl1 in the adult dentate gyrus SGZ in Type-1 cells, a population of cells defined as stem cells since they maintain the ability to generate new neurons, at least in young adult mice [18]. However, our previous efforts to determine the dynamics of Ascl1^+^ progenitor cell development defined a population of cells that transitioned to postmitotic, NeuN^+^ cells within 30 days [7]. As this previous work used a transgenic mouse containing a BAC with the Ascl1 coding region replaced by CreER™, we reexamined this issue with an *Ascl1^CreERT2^* knock-in mouse strain where CreER^T2^ replaced endogenous Ascl1 (Fig. 2A) such that CreER^T2^ is restricted to Ascl1 expressing cells (Fig. 3E–E′). TAM was administered to *Ascl1^CreERT2/+^;R26R^YFP/YFP^* mice 6–7 weeks old, and the Ascl1 lineage was analyzed 7, 30, and 180 days post-TAM, utilizing YFP expression from the Cre reporter [12]. In the SGZ 7 days post-TAM, 49% of YFP^+^ cells were Sox2^+^ early progenitors, with a subset of these (12%) presenting Type-1 cell morphology or labeling for GFAP (Fig. 2C–E′). Furthermore, although Ascl1 itself rarely co-localizes with NeuroD1, 53% of YFP^+^ cells were NeuroD1^+^ identifying them as Type-2b or 3/immature neurons (Fig. 2F–F′), and implying that cells expressing CreER^T2^ 7 days prior have transitioned to later stages within the lineage. 7 days post-TAM no YFP^+^ cells co-labeled with NeuN, a marker of mature neurons (Fig. 2B). However, 30 days post-TAM, the population continued to mature, such that 26% of YFP^+^ cells were NeuN^+^ granule neurons (Fig. 2J–J′). Notably, even after 30 days post-TAM many YFP^+^ cells expressed markers of progenitor cells, with 29% Sox2^+^ and 36% NeuroD1^+^, and with 16% clearly showing Type-1 cell morphology and expressing GFAP (Fig. 2G–I′). This result is in contrast to that seen when marking only Type-2 cells, which would all have transitioned to NeuN^+^ neurons 30 days post-TAM [7].

To determine the fate of the marked cells over longer periods, we examined brains 180 days post-TAM. Neurogenesis in the hippocampus declines dramatically between 12 and 34 weeks of age [19], illustrated here by fewer cells expressing progenitor markers (NeuroD1, Doublecortin (DCX), and Ki67; Fig. 2O–V). Notably, there is no obvious loss of Sox2^+^ cells, suggesting Sox2 may mark quiescent Type-1 cells that are only rarely dividing in aged brains. 180 days post-TAM, 65% of Ascl1 lineage cells marked by YFP were NeuN^+^ granule neurons (Fig. 2N–N′). Strikingly, 25% of the YFP^+^ cells continued to express Sox2, including 10% with Type-1 cell morphology and expression of GFAP (Fig. 2K–M′). Consistent with the near absence of NeuroD1 and DCX at this age (∼34 weeks old), no YFP^+^ cells co-labeled with these markers (Fig. 2B).

These Ascl1 lineage results combined with localization of Ascl1 in Nestin::GFP^+^/GFAP^+^ cells in the adult SGZ demonstrate that Ascl1 is present in a population of cells with persistent neurogenic potential beyond that expected from a population of transit amplifying Type-2 cells. Indeed, these findings with *Ascl1^CreERT2^* are comparable with those found in a similar paradigm with *Nestin-CreER^T2^* or *Gli1-CreER^T2^* which was shown to mark Type-1 stem-like cells in the adult SGZ [11], [20].

### Ascl1 marks a population of cells in the adult SVZ that have long term neurogenic potential

To determine if Ascl1 also defines a population of cells with long term potential to generate neurons in the SVZ, we examined Ascl1 expression in the SVZ of *Nestin::GFP* mice and the dynamics of the adult-generated Ascl1 lineage cells in the SVZ, rostral migratory stream (RMS), and olfactory bulb in *Ascl1^CreERT2/+^;R26R^YFP/YFP^* mice. As seen in the SGZ (Fig. 1), SVZ Ascl1^+^ cells were heterogeneous in fluorescent intensity, with cells expressing high versus low levels of Ascl1 immunoreactivity (Fig. 3A–B′, D), with the putative stem (B cells) and progenitors (C cells) readily defined by published criteria (B cells GFAP^+^/Nestin::GFP^+^; C cells GFAP^−^/Nestin::GFP^+^). In contrast to the SGZ, a much greater proportion of Ascl1^High^ and Ascl1^Low^ expressing cells in the SVZ were C versus B cells. Notably, no Ascl1^High^ B cells were present in the RMS, although many were C cells (Fig. 3C–C′, D). Indeed, the majority of Ascl1 cells were progenitors or C cells (Fig. 3D) consistent with previous reports [21]. This is also consistent with conclusions from lineage tracing studies using the BAC *Ascl1::CreER* transgenic mouse which showed 30 days post-TAM all Ascl1 lineage marked cells had differentiated to neurons in the olfactory bulb [7]. However, the presence of Ascl1 in a subpopulation of SVZ B cells suggests that Ascl1 may be marking a stem cell population, or at least a population with long term neurogenic potential in this region of the adult brain.

To determine the developmental dynamics of these Ascl1 cells in the adult SVZ, we used the same paradigm as described above for the hippocampus using the *Ascl1^CreERT2/+^* knock-in mouse strain. Adult (P50) *Ascl1^CreERT2/+^*;*R26R^YFP/YFP^* mice were treated with TAM for 5 consecutive days. At 7 days post-TAM, YFP^+^ cells co-expressing Sox2 or DCX were found in the SVZ or along the RMS, whereas no cells co-expressing NeuN were detected (Fig. 3F–H, O–P′, data not shown). At 30 days post-TAM, YFP^+^ cells co-expressed NeuN in the olfactory bulb, demonstrating labeled cells are migrating and maturing into neurons (Fig. 3I, R–R′). Notably, many YFP^+^ cells in *Ascl1^CreERT2/+^;R26R^YFP/YFP^* mice still remained in the SVZ and RMS and expressed Sox2, DCX, or the proliferation marker Ki67 30 days or even 180 days after initial *Ascl1* expression (Fig. 3J–N, Q–Q′, S–T′, data not shown). This result demonstrates that *Ascl1* expressing cells in the adult SVZ are not just transit amplifying neural progenitors, but at least some of these cells have long-term (180 days) potential to generate neurons, implying they represent a subset of self-renewing neural stem cells in this region of the brain.

## Discussion

Ascl1 is an essential regulator in multiple regions of the embryonic nervous system in the balance of whether a cell is maintained as a progenitor or whether it differentiates(for review see [5]). It has also recently been shown to be a critical component in the cocktail, along with Pou3f2 and Mytl1, for directly reprogramming fibroblasts to neurons [3], and it has aberrant expression in neural tumors such as glioblastoma [22], [23]. Given that Ascl1 is transiently expressed in adult neurogenic niches [7], defining the cell types in these lineages that express Ascl1 provides insight into their molecular identity and in the process of adult neurogenesis. We show here that Ascl1 is present in populations that continuously generate olfactory bulb neurons from the adult SVZ. This is seen in the generation of new YFP-labeled neurons up to 180 days after labeling in the adult brain of *Ascl1^CreERT2^* mice. In the SGZ, cells from the Ascl1 lineage also generate neurons over extended periods (30 days) but as the animal ages and neurogenesis decreases, so does the generation of new neurons from *Ascl1* marked cells. However, there is a persistent population of labeled cells with a neural stem cell phenotype up to 180 days post-TAM. Together these results imply that Ascl1 is present in at least a subset of self-renewing, neuron-generating cells. This conclusion is in contrast to our previous fate mapping studies using a BAC transgenic *Ascl1::CreER™* mouse, where essentially all lineage marked cells differentiated to mature neurons within 30 days in the SVZ and the SGZ, which suggested that in this paradigm Ascl1 lineage cells are restricted to the transit amplifying populations [7]. The *Ascl1^CreERT2/+^* knock-in mouse used here more accurately recapitulates the patterns and timing of *Ascl1* expression than that seen in the BAC transgenic mouse based on comparison of *Ascl1* and *Cre* in situ patterns in multiple tissues at multiple stages in the two mouse strains (Fig. 3E–E′ and data not shown), and on the detection of Ascl1 lineage cells in the retina, olfactory epithelium, and lung in the knock-in model not labeled with the BAC transgenic model (EJK and JEJ, unpublished). While *Ascl1^CreERT2/+^* knock-in mice have only one copy of *Ascl1* in contrast to the BAC model, no phenotype in *Ascl1* heterozygous animals has been reported. Results here show the *Ascl1^CreERT2/+^* line is labeling a population of cells at an earlier stem-like progenitor stage than was previously appreciated.

Our placement of Ascl1 in a subset of GFAP^+^ cells in the SVZ is consistent with a report by Pastrana et al. who characterized expression of GFAP and Ascl1 in EGFR^+^ cells isolated from adult mouse SVZ [24]. In this study, EGFR^+^ cells defined two populations, with 37% of “activated stem cell astrocytes” (GFAP^+^/EGFR^+^) expressing Ascl1 and a greater proportion of “transit amplifying cells” (GFAP^−^/EGFR^+^) expressing Ascl1 [24]. Notably, this study found that Ascl1 levels were lower in GFAP^+^ cells than in GFAP^−^ cells. This is consistent with our results, and supports the conclusion that *Ascl1^CreERT2^* is marking cells that endogenously express Ascl1, even at low levels. The ability of the GFAP^+^/EGFR^+^ cells to generate neurospheres [24], combined with the long term potential of *Ascl1* lineage cells to generate olfactory bulb neurons, implies Ascl1 is present in neural stem cells in the SVZ.

In the SGZ, the *Ascl1^CreERT2^* marked cells have a more limited potential to generate neurons than that seen in the SVZ. However, this limitation may reflect the age-related decrease in SGZ neurogenesis [19], and our data remain consistent with the ability of Ascl1 to mark neural stem cells in both the SGZ and SVZ. In our lineage tracing studies presented here, two population of marked cells were evident in the mice 180 days post-TAM: dentate gyrus granule neurons (NeuN^+^, presumably integrated into hippocampal circuitry), and SGZ Type-1 cells (GFAP^+^ with radial glial morphology). Whether these Type-1 YFP^+^ cells in 34 week-old mice have the potential to generate new neurons in response to stimulation is not known. The TAM-inducible Cre lineage marking paradigm used here is unable to distinguish whether these remaining cells are a distinct population in the SGZ that did not participate in generating the neurons earlier, or whether they are the same stem cell population that is now quiescent. Nevertheless, the Ascl1-lineage marked cells retain the ability to generate new neurons at least for 30 days in the SGZ of the hippocampal dentate gyrus, implying they also represent a subset of neural stem cells.

Ascl1 function in neural development includes a major role in the timing of neuronal differentiation in a balance with Notch signaling. As a member of the proneural subclass of bHLH factors, emerging models would place low Ascl1 activity at early stages to give tone to Notch-regulated progenitor maintenance [17]. Additionally, differing Ascl1 levels could result from unequal distribution of Ascl1 during asymmetric divisions. Once higher levels of Ascl1 activity are reached, cells are committed to neuronal differentiation. Demonstrating the role for Ascl1 in supporting the maintenance of GFAP^+^ neural stem-like populations must await analysis of an *Ascl1* conditional knockout. The expression characteristics of Ascl1 shown here and in Pastrana et al., combined with our in vivo lineage tracing over time, clearly place Ascl1 in cells with long-term neurogenic potential in the adult brain, a population previously believed to be without *Ascl1* expression, and may reflect the interplay of Ascl1 with Notch signaling to regulate the dynamic equilibrium between stem cell maintenance and differentiation [15], [25], [26].

## Materials and Methods

### Ethics Statement

This study was carried out in strict accordance with the recommendations in the Guide for the Care and Use of Laboratory Animals of the National Institutes of Health. All procedures used were approved by the University of Texas Southwestern Medical Center Institutional Animal Care and Use Committee APN 2007-0065. All efforts were made to minimize suffering.

*Ascl1^CreERT2^* knock-in mice were generated by replacing the *Ascl1* coding region with *CreER^T2^* [8] and Frt-Neo-Frt cassettes. The targeting strategy was the same used to generate *Ascl1^GFP^* knock-in mice [9]. The endogenous ATG was replaced by a short sequence containing a PacI site and a consensus Kozak site. The correct targeting event was identified by Southern analysis of EcoRI digested DNA using 5′ and 3′ probes. After obtaining germ line transmission in the *Ascl1^CreERT2-Frt-Neo-Frt^* mice, they were crossed with FLPe mice [10] to remove the neomycin cassette resulting in *Ascl1^CreERT2^* mice.

For PCR genotyping, the following primers were used: 5′-AAC TTT CCT CCG GGG CTC GTT TC-3′ (Sense Ascl1 5′UTR) and 5′-CGC CTG GCG ATC CCT GAA CAT G-3′ (Anti sense Cre) giving a PCR product of 247 bp. Tamoxifen (TAM) induction of Cre recombinase was accomplished by intraperitoneal injection of *Ascl1^CreERT2/+^;R26R^YFP/YFP^* postnatal day 50 (P50) mice with 180 mg/kg/day TAM (Sigma, T55648) in sunflower oil on five consecutive days. Brains were harvested at the times specified after TAM and processed as described [7], [11]. *R26R^YFP^* and *Nestin::GFP* mice have been previously described [12], [13].

For immunofluorescence staining, free floating sections or sections mounted on slides were incubated in the appropriate dilution of primary antibody in PBS/3% donkey (or goat) serum/0.2% NP-40 (or 0.2% Triton X-100), followed by appropriate secondary antibody conjugated with AlexaFluor 488, 568, or 594 (Molecular Probes). Mouse monoclonal antibodies used were: Ascl1 (1∶750, RDI Fitzgerald, 10R-M106B), NeuN (1∶1000, Chemicon, MAB377), GFAP (1∶400, Sigma, G3893). Rabbit polyclonal antibodies used were: GFP (1∶500, Molecular Probes, A6455), GFAP (1∶500, DAKO, Z0334), Ki67 (1∶500, Neomarker), Sox2 (1∶2000, Millipore). Goat polyclonal antibodies used were: DCX (1∶200, Santa Cruz) and NeuroD1 (1∶200, Santa Cruz). Chick GFP (1∶500, Aves Lab) was also used. Confocal imaging was carried out on a Zeiss LSM510 confocal microscope. Ascl1^+^ fluorescence intensity levels were classified as high or low using ImageJ and setting a threshold of pixel intensity for Ascl1^Low^ (314–599 units) and Ascl1^High^ (>600 units). For cell number counts, three *Nestin::GFP* mice were analyzed to place Ascl1^+^ progenitors in the adult neural stem cell lineage. For in vivo genetic tracing experiments using the *Ascl1^CreERT2^* knock-in line, at least two *Ascl1^CreERT2/+^;R26R^YFP/YFP^* mice per each harvest time point (7, 30, or 180 days post-TAM) were used. For co-localization data with each stage-specific marker, 150–500 YFP^+^ cells per animal were counted.
